# Alteration of intestinal microflora by the intake of millet porridge improves gastrointestinal motility

**DOI:** 10.3389/fnut.2022.965687

**Published:** 2022-08-22

**Authors:** Ying Chen, Rong Zhang, Jialiang Xu, Qing Ren

**Affiliations:** ^1^School of Light Industry, Beijing Technology and Business University, Beijing, China; ^2^Beijing Advanced Innovation Center for Food Nutrition and Human Health, Beijing Technology and Business University (BTBU), Beijing, China; ^3^Xinjiang Second Medical College, Karamay, China

**Keywords:** foxtail millet, intestinal microflora, constipation, prebiotic, gastrointestinal motility

## Abstract

Foxtail millet (*Setaria italica*) has a long history of treating gastrointestinal ailments in China; however, little is known about the functional mechanism driving its therapeutic effects. The primary edible form of millet is porridge. This study investigates the effects of millet porridge on diphenoxylate-induced constipation and intestinal microflora in mice. Fifty mice were randomly divided into five groups: normal control group, constipation model group, and low-dose, medium-dose, and high-dose millet porridge groups. After 14 days of millet porridge gavage, constipation was induced and measured. The results showed that millet porridge prevented constipation by increasing the water content of feces, shortened the time of the first melena defecation, promoted gastric emptying, and improved the rate of gastrointestinal propulsion. Millet porridge also dose-dependently increased levels of *Bifidobacterium* and *Lactobacillus* and decreased levels of *Escherichia coli*, *Enterococcus*, and *Bacteroides* in the intestine. These results show that millet porridge could accelerate intestinal motility and change the proportions of intestinal flora and that it has a potent prebiotic effect.

## Introduction

Gut motility is influenced by several factors, including the nervous and immune system, mucus secretion, gastrointestinal microbiota, and fermentation products. Chronic constipation is a typical symptom of gut motility disorder (1). Epidemiological studies show that the global prevalence of constipation is 14% (2). This rate modestly increases with age and is almost two times higher in women than in men (17.4 vs. 9.2%) (3). Constipation seriously affects quality of life, which poses a considerable economic and medical burden, more so than type 2 diabetes or irritable bowel syndrome, and affects work productivity (4). Reports have also demonstrated that constipation is related to mood disorders such as anxiety and depression (5, 6).

Foxtail millet has been cultivated for 8,000 years in China and is primarily distributed in northern China and India (7). It is also known as Italian/German millet worldwide (8, 9). Traditional Chinese medicine considers millet beneficial for nausea and dysentery treatment. Millet porridge helps coordinate the intestines and stomach, tonify deficiency, and can stimulate the appetite. It also prevents indigestion and assists in treating gastric ulcers and digestive ulcers (10). Nutritional research on foxtail millet has revealed many health benefits, including blood-sugar-lowering effects (11), and anti-oxidative (12), antifungal (13), antihypertensive (14), and anti-inflammatory behavior (15). The positive effects of consuming foxtail millet or foods on human health have garnered significant research attention.

In recent years, studies have demonstrated that intestinal microbiota play a key role in health and that microbiota affect various physiological activities of the host, including gastrointestinal motility (16–19). Diet is also a key factor determining the composition of human microbiota (20, 21). Therefore, we aimed to elucidate how millet porridge affects gut motility and the intestinal environment in mice.

## Materials and methods

### Materials

Foxtail millet was provided by the Hebei Academy of Agricultural Sciences and was mixed in distilled water (1: 20 ratio) and boiled for 20 min at 100°C to make porridge. The millet porridge was made into freeze-dried powder for laboratory analysis and mouse experiments. Analytical results of the nutritional content of millet porridge are provided in Table 1. The samples were analyzed using standard method of analysis of nutrients in foods as follows: the AOAC Official method 920.39, 984.13, 942.05, 934.01, and 962.09 were used to determine the crude fat, protein, ash, moisture, and crude fiber content, respectively. Total carbohydrates were determined by difference method [100–(Crude Fat + Protein + Ash + Moisture)]. The iodine binding assay with dual wavelength (λ 620 nm and λ 510 nm) measurement reported by Zhu et al. (22) was used to determine the amylose content. Amylopectin was calculated by difference method (100 – Amylose). All chemical reagents used in this work are analytically pure.

**TABLE 1:** The nutritional content of millet porridge.

Composition	Crude fat	Protein	Ash	Moisture	Carbohydrates	Crude fiber	Amylose	Amylopectin
Content (%)	3.74 ± 0.68	10.3 ± 0.52	0.90 ± 0.06	6.56 ± 0.10	78.50 ± 1.11	11.23 ± 0.38	23.86 ± 1.02	76.14 ± 1.02

### Animals

Healthy Kunming male mice (18–22 g body weight) were purchased from Weitonglihua (Beijing, China), and were housed in the Animal Experiment Center of Beijing University of Chinese Medicine. All mice were maintained in laboratory cages under a 12-h light/dark cycle at a temperature of 23 ± 3°C and 35 ± 5% humidity with no pathogens and were provided with unlimited food (regular chow). All experimental procedures involving mice were approved by Ethics Committee of Beijing University of Chinese Medicine.

### Effects of millet porridge on gastrointestinal motility

#### Induction of constipation model in mice

Constipation was induced in mice according to previous method, with minor modifications (23, 24). Briefly, mice were orally administered with diphenoxylate (10 mg/kg of body weight), while the normal control mice were administered with normal saline. If the paste propulsion rate of mice was significantly declined, defecating time of first black feces was distinctly prolonged, and total weight and number of black feces within 6 h were both evidently decreased after diphenoxylate treatment, in comparison with control mice, it could be concluded that the constipation model of mice was established successfully.

#### Defecation test

The experiments were performed according to the literature (25). Fifty mice were randomly divided into the normal control group, constipation model group, and low-dose, medium-dose, and high-dose millet porridge groups (*n* = 10 mice each). According to international standards dictating the recommended daily intake of cereals for a healthy adult and after accounting for differences between species, mice should be given 10 times the human dose. The low-dose, middle-dose, and high-dose groups were given 3, 6, and 12 g/kg of their body weight of millet porridge daily, respectively. In addition, the normal control group and constipation model group were given a normal saline solution daily for 14 consecutive days. All treatments were administered intragastrically. On the fifth day, 1 h after routine gavage, each group was orally administered 10% activated carbon suspension. The defecation time was recorded when black feces were first excreted. All feces granules were collected within 6 h. Finally, the weight and water content of the feces of each mouse were measured.

After 14 days of gavage of millet porridge, feeding was withheld from all mice for 24 h, but they were allowed *ad libitum* to water. Diphenoxylate (10 mg/kg body weight) was administered to the model group, and the millet porridge treated groups to induce constipation. Equivalent distilled water was given to the control group. Thirty minutes after diphenoxylate treatment, the test groups were gavaged with millet porridge, and the normal control and constipation model groups were given normal saline. After another thirty minutes, 10% activated-carbon suspension solution (20 ml/kg) was orally administered to all groups. The defecation time was then recorded, and the weight and water content of the feces were measured as previously described.

#### Gastric emptying and intestinal propulsion test

Another 50 male mice were used for the defecation test and were divided into the same five groups (*n* = 10 mice each). After 14 days of administering millet porridge, food (but not water) was withheld from the mice for 24 h. The model group and the millet porridge treated groups induced constipation by diphenoxylate. Equivalent distilled water was administered to the control group. Thirty minutes later, a volume of 0.8 ml semi-solid paste was orally administered, and the paste was weighed and recorded as WSSP. After twenty minutes, the mice were anesthetized and sacrificed. The pylorus and cardia were ligated, and the stomach was removed, weighed, and recorded as the total weight (TW). The stomach was then cut along the greater curvature to remove the contents and was weighed again and recorded as the net weight (NW). The gastric emptying ratio was calculated by the following equation:


Gastric⁢emptying⁢ratio=[1-(TW-NW)/WSSP]× 100%

The small intestine of each mouse was also removed to measure the intestinal propulsion rate. The total length (TL) of the small intestine (from the pylorus to the cecum) and the length (L) from the pylorus to the front end of the paste were recorded. The intestinal propulsion ratio was calculated by the following equation:


Intestinal⁢propulsion⁢ratio=(L/TL)× 100%

### Effects of millet porridge on intestinal microflora

#### Identification and quantification of intestinal microbiota

One hundred and twenty male Kunming mice were divided into four groups: control group, low-dose, medium-dose, and high-dose millet porridge groups (*n* = 10 mice each). All groups were treated for 28 days, then free-fed for another 7 days. Fresh feces were collected from the rectum on the 14th, 28th, and 35th day of the experiment, the colon and cecum were removed, and the contents were harvested (10 mice for each group). A total of 200 mg of fresh feces, colon, or cecal content was suspended in 1.8 ml of phosphate-buffered saline with a glass bead and shaken with TissueLyser. Two dilutions of homogenate were then prepared, and 100 μl of each dilution was inoculated into the selective medium. MacConkey agar for *Escherichia coli*, PSE Enterococcus Selective agar for that organism, with aerobic culture for 24 h. BDS medium for *Bacteroides*, BBL agar for *Bifidobacterium*, *Lactobacillus* Selection agar for that organism, and plates were incubated for 48 h in an anaerobic cabinet. For each culture, the number of bacteria was determined by plating. The colonies selected in each medium were identified by 16S rDNA PCR (26). The primers were 5′-AGAGTTTGATCCTGGCTCAG-3′ and 5′-GGTTACCTTGTTACGACTT-3′ for forward and reverse primers, respectively.

#### PCR-denaturing gradient gel electrophoresis

PCR-denaturing gradient gel electrophoresis (DGGE) was performed as previously described (27), with a few modifications to optimize the protocol. The profiles were analyzed using Quantity One and analyzed by UPGMA cluster analysis.

The Shannon diversity index (*H*), richness (*S*), and evenness (*E*) were used to further analyze the diversity of intestinal microflora:


H=-∑(Pi)⁢⁢(lg⁢Pi)

E=H/H⁢max=H/1nS

Pi, the proportion of species *i* relative to the total number of species; *S* is the total number of colored bands for each sample.

### Statistical analysis

Statistical analysis was performed using GraphPad Prism (Version 5, GraphPad Software Inc., San Diego, CA, United States). The data were expressed as the mean ± SD. One-way analysis of variance (ANOVA) was used to compare different groups. *P* < 0.05 was considered statistically significant.

## Results

### Effects of millet porridge on gastrointestinal motility

The effects of millet porridge on the feces characteristics of mice in the normal and constipation models were determined. After 5 days of gavage, the high- and middle-dose millet porridge groups significantly decreased the defecation time and increased feces weight produced at 6 h (Table 2). However, there was no significant difference in the water content of the feces between the groups. Next, we employed the constipation model. In the model group, there was a significantly longer time until the first black feces than in the control group, indicating that the constipation model was successfully induced with diphenoxylate (Table 2). In the millet porridge groups, the time to first black feces was less than in the model group; middle- and high-dose millet porridge significantly decreased the defecation time. Millet porridge groups also significantly increased the weight and moisture content of the feces. These results suggest that millet porridge has a laxative effect and can help induce defecation.

**TABLE 2:** Effects on the first defecation time, weight, and water content of feces in normal and constipated mice.

Group		Millet porridge dose (g/kg body weight per day)	First defecation time (min)	Feces weight (mg)	Feces water content (%)
Normal	Control	0	94 ± 12*^a^*	200 ± 12*^a^*	47.50 ± 2.5
	High	12	62 ± 22*^b^*	270 ± 12*^b^*	48.21 ± 2.3
	Middle	6	63 ± 18*^b^*	250 ± 12*^b^*	44.78 ± 3.5
	Low	3	87 ± 12*^a^*	230 ± 15*^ab^*	45.93 ± 3.3
Constipated	Control	0	84 ± 11*^a^*	380 ± 15*^c^*	45.22 ± 2.8*^b^*
	Model	0	129 ± 22*^e^*	230 ± 12*^a^*	35.42 ± 2.4*^a^*
	High	12	110 ± 10*^cb^*	370 ± 14*^c^*	43.51 ± 2.3*^b^*
	Middle	6	118 ± 21*^cd^*	370 ± 16*^c^*	42.16 ± 2.1*^ab^*
	Low	3	122 ± 12*^de^*	320 ± 12*^b^*	45.31 ± 3.4*^b^*

^*a–e*^Means that different letters in the same column indicate a significant difference (*P* < 0.05) between them.

We next determined the effect of millet porridge on the gastric emptying rate in constipated mice. Following diphenoxylate treatment, the gastric emptying ratio significantly decreased compared with the control (*P* < 0.01). As shown in Supplementary Figure 1A, compared with the model group, the gavage of millet porridge significantly accelerated the gastric emptying rate, an effect that was dose-dependent. High-dose millet porridge was able to recover to control group levels.

Next, we determined the intestinal propulsion rate of mice (Supplementary Figure 1B), which was significantly higher than in the model group and depended on the dose. This suggests that the gastrointestinal propulsive rate was increased by millet porridge, which can effectively increase movement in the small intestine.

### Effects of millet porridge on intestinal microflora

**FIGURE 1:**
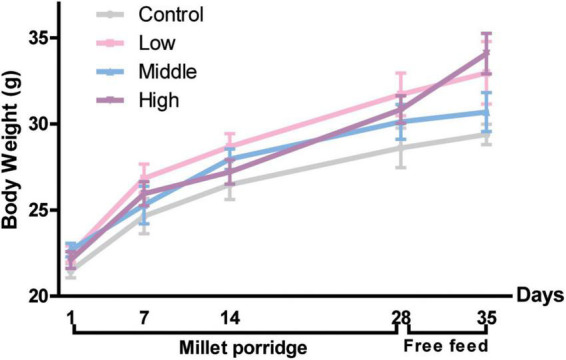
Body weight of mice. The data are means ± SD. Male Kunming mice were gavaged with millet porridge (3, 6, 12 g/kg body weight each day in the low-, middle-, and high-dose group, respectively) for 28 days, then free-fed for another 7 days.

Given that microbiota affects the gastrointestinal motility of the host, we assessed whether millet porridge induces physiological functions due to fermentation, which could increase probiotics in the intestine. No significant changes in body weight were observed between the four groups during the experiment, indicating that millet porridge does not negatively affect the body weight or growth of mice (Figure 1).

**FIGURE 2:**
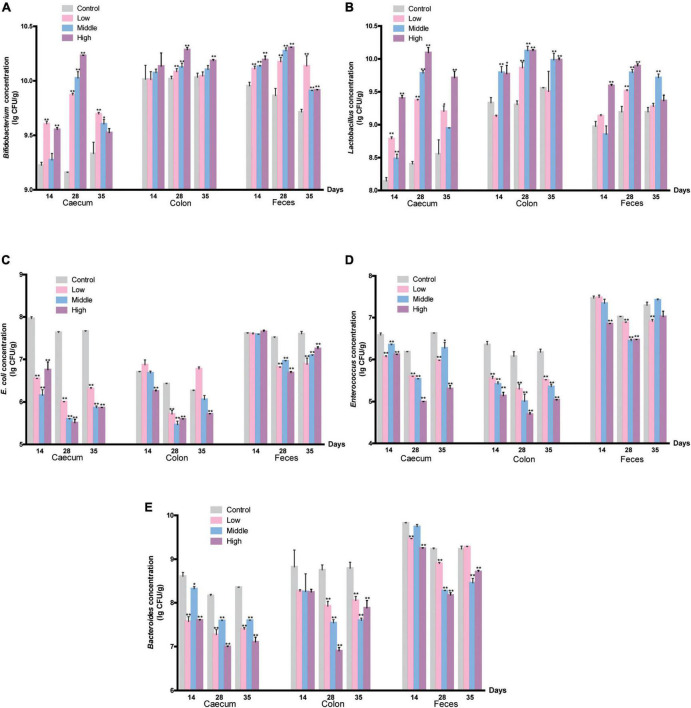
The amount of intestinal bacteria. **(A)** *Bifidobacterium*; **(B)** *Lactobacillus*; **(C)** *E. coli*; **(D)** *Enterococcus*; **(E)** *Bacteroides*. Data are presented as mean ± SD (*n* = 10 for each group). *P < 0.05, and **P < 0.01 represent a significant difference from the control group.

We identified *Bifidobacterium, Lactobacillus, E. coli*, *Enterococcus*, and *Bacteroides* in the cecum, colon, and fresh feces of mice by selective culture and 16S rDNA PCR. The number of each type of bacteria was counted. *Bifidobacteria* markedly increased on the 14th day, and as the gavage time increased, millet porridge dose-dependently increased the number of *Bifidobacteria* (Figure 2A). *Lactobacillus* in the cecum, colon, and fresh feces were significantly higher than in the control group on the 28th day, and the high-dose group was significant (Figure 2B). One week after gavage ended, the number of *Bifidobacterium* and *Lactobacillus* decreased in the tested groups but was still higher than in the control group. These results indicate that millet porridge promoted the growth of *Bifidobacterium* and *Lactobacillus* in the intestinal tract.

In addition, the abundance of *E. coli* in the cecum significantly decreased in millet porridge-treated groups on the 14th day (*P* < 0.05) (Figure 2C). As the gavage time increased, the number of *E. coli* in the cecum, colon, and fresh feces all significantly decreased in the three tested groups compared with the control group (Figure 2C). Similar results were found in changes of *Enterococcus* and *Bacteroides* (Figures 2D,E). The number of *Enterococcus* and *Bacteroides* in the colon and feces significantly decreased during millet porridge gavage, an effect that was dose-dependent. One week after gavage ended, the number of the three pathogenic bacteria recovered in all tested groups but was still lower than in the control group. Altogether, these data indicate that millet porridge decreased the abundance of pathogenic bacteria such as *E. coli, Enterococcus*, and *Bacteroides* but promoted the proliferation of probiotics such as *Bifidobacterium* and *Lactobacillus*.

### Phylogenetic diversity of main bacteria

**FIGURE 3:**
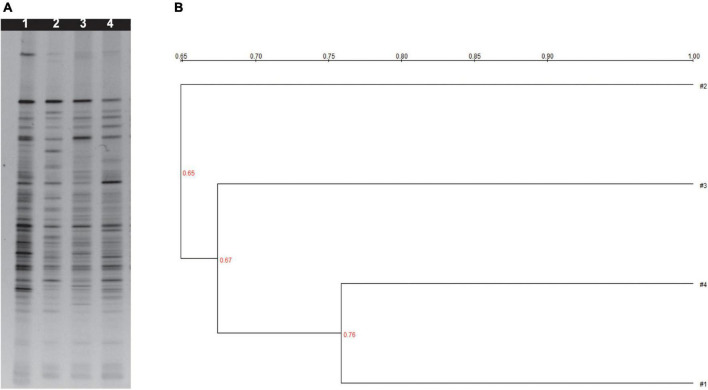
**(A)** Denaturing gradient gel electrophoresis profiles from bacteria of fresh mice feces. **(B)** Cluster analysis analyzing Dice similarity index of DGGE profiles. 1 was treated with control; 2 was treated with 12 g/kg millet porridge; 3 was treated with 6 g/kg millet porridge; and 4 was treated with 3 g/kg millet porridge.

PCR-denaturing gradient gel electrophoresis analysis with universal primers targeting the V3 region of 16S rRNA was used to analyze the main intestinal microflora in the feces of mice given millet porridge (3, 6, and 12 g/kg) or the control (Figure 3A).

Differences in the level of brightness and the number of the bands were observed in the DGGE profiles, which were also used to identify the diversity index (*H*), richness (*S*), and evenness (*E*) (Supplementary Table 1). Millet porridge gavage significantly increased the *H*, *S*, and *E* in the feces compared with the control treatment (*P* < 0.05), while the middle- and high-dose groups had the most significant effect.

Clustering analysis based on the Dice coefficients was visualized; three primary clusters were identified in the feces dendrograms (Figure 3B). The first cluster is the low-dose millet porridge and the control group, the second cluster is the middle-dose treatment, and the third cluster is the high-dose treatment. The results demonstrate that middle- and high-dose millet porridge treatments have a greater effect on intestinal microflora diversity.

## Discussion

It has been demonstrated that diphenoxylate acts directly on intestinal smooth muscle and inhibits peristalsis, thus delaying intestinal contents of advancing speed and reducing bowel movements (28, 29). In this study, diphenoxylate induced a significant decline in the paste propulsion rate, prolonged defecating time of the first black feces, and reduced the black fecal granule number and weight, suggesting that the constipation model in this study was successfully established. Our study found that millet porridge could ameliorate diphenoxylate-induced constipation and improve intestinal motility in mice.

Gastrointestinal motility disorders are primarily marked by sluggish gastric release and gastrointestinal motility, which negatively affect digestive functioning. Constipation is a typical symptom of a gut motility disorder. As quality of life and imbalanced diets have become more prevalent, constipation has become more common in developing countries, such as China. Accumulating evidence has demonstrated that the composition of the colonic mucosal microbiota is different between constipated patients and controls. For example, adults with functional constipation have significantly decreased numbers of *Bifidobacteria* and *Lactobacilli* and increased *Bacteroidetes* in feces samples compared with controls. The colonization of a certain pathogen-free microbiota can normalize small-bowel migrating motor complexes (30), while *Lactobacillus acidophilus*, *Bifidobacterium bifidum*, or *Clostridium tabificum* colonizing germ-free rats can normalize small-bowel migrating motor complexes and gut transit time. *E. coli* colonization inhibited intestinal myoelectric activity (31). Thus, modifying the gut luminal environment could impact gut motility and secretion and help patients with constipation.

Foxtail millet has long been considered healthy food that promotes gastrointestinal health in China. Lin showed that millet has a great beneficial effect on gastric mucosal barrier protection (10). Several studies reported that millet exhibit significant protective effects on the viability of *Lactobacillus* (32, 33). Porridge is the main form of edible millet, and there is little published research on millet porridge. Our study found that millet porridge could dose-dependently increase levels of *Bifidobacterium* and *Lactobacillus* and dose-dependently decrease levels of *E. coli*, *Enterococcus*, and *Bacteroides* in the intestine.

The DGGE profiles suggest that millet porridge increased the diversity of the intestinal microflora. While PCR-DGGE is unable to identify bacterial species, it can help assess the diversity of intestinal microbiota. Changes in the bacterial community can be observed through DGGE profiles by highlighting changes in the main species of bacteria. Recent research has used PCR-DGGE to assess how dietary supplements affect the intestinal microbiota of animals (34–36). The number and intensity of DGGE bands could reflect the complexity and diversity of intestinal flora under different treatments. Our data showed that oral gavage with millet porridge at 6 and 12 g/kg had a significantly higher diversity of intestinal microflora than those treated with the control. As such, millet porridge can improve intestinal health in mice by widening the diversity of intestinal microflora. Given the benefit of millet porridge to intestinal microflora, it is potential to conduct the research as novel prebiotics.

Dietary fiber is considered beneficial on health promotion through the improvement of the balance of intestinal bacterial flora (37, 38). We found that millet porridge contained 23.86% amylose, which is an important factor for formation of resistant starch (RS). Thermal treatment includes cooking millets beyond gelatinization temperature and gradually cooling for retrogradation can increase RS (39), the millet porridge used in our trials could contain numerous RS and display an important role. As a soluble dietary fiber, RS has been described as having a laxative effect, which could be due to fermentation, and increases probiotics found in the intestine (40). And one study reported that millet dietary fiber enhanced short chain fatty acids production by fecal probiotic bacteria fermentation (41). In fact, 52 DGGE fragments were isolated and sequenced, and we found that the dominant bacteria induced by millet porridge include *Butyrivibrio*, *Desulfovibrio*, and *Clostridium cellulolyticum*. These bacteria can degrade cellulose and hemicellulose, and increase the concentration of short chain fatty acid, such as acetate and butyrate in the colon (42–44). Therefore, we speculate that the millet porridge help regulate intestinal flora, promote intestinal motility, and accelerate gastric emptying. Further studies about millet porridge will involve components function analysis and assess the mechanisms regulating intestinal microbes and physicochemical markers for constipation.

## Conclusion

Our study demonstrated that millet porridge treatment prevented diphenoxylate-induced constipation in mice, promoted intestinal motility (which decreased the time to the first black feces), and increased the water content of the feces, wet weight, gastric emptying, and the intestinal propulsion rate. Millet porridge also increased the abundance of some presumably beneficial intestinal bacteria (e.g., *Bifidobacterium* and *Lactobacillus*) in the intestinal tract and decreased the abundance of bacteria with pathogenic potential (e.g., *E. coli*, *Enterococcus*, and *Bacteroides*). These changes are correlated with symptoms of constipation. One limitation of this study is that we did not investigate the mechanisms underlying intestinal microbial regulation of physicochemical indexes of constipation. These data suggest that millet porridge accelerates intestinal motility and regulates intestinal micro-ecology and could be a natural and effective method of treating constipation.

## Data availability statement

The original contributions presented in this study are included in the article/Supplementary material, further inquiries can be directed to the corresponding author.

## Ethics statement

This animal study was reviewed and approved by the Ethics Committee of Beijing University of Chinese Medicine.

## Author contributions

YC and QR designed the study. RZ performed the experiment. JX analyzed the data. QR revised the manuscript. All authors contributed to the article and approved the submitted version.

## Supplementary material

Supplementary Figure 1 Effect of millet porridge on gastric emptying **(A)** and intestinal propulsion **(B)**. Diphenoxylate was used to induce the constipation model. The data are means ± SD (*n* = 10 for each group), ^##^*P* < 0.01 vs. control; **P*<0.05, ***P* < 0.01 vs. Model.
