# Supplementary material for: Alteration of intestinal microflora by the intake of millet porridge improves gastrointestinal motility
Source: Front Nutr. 2022 Aug 22;9:965687. doi: 10.3389/fnut.2022.965687 (PMC9442030; doi:10.3389/fnut.2022.965687)
Supplement: Supplementary file 1 [file Table_1.pdf]

Supplementary Table 1. Shannon diversity index, richness and evenness of PCR-DGGE profiles of feces samples of mice.

**Groups**	**Control**	**Millet porridge**		
		3 g/kg	6 g/kg	12 g/kg
**Diversity index (*H*)**	2.55±0.10[a]	2.90±0.05[c]	3.38±0.16[b]	3.33±0.13[b]
**Richness (*S*)**	38.33±1.15[a]	39.33±0.13[ab]	45.33±2.08[c]	42.67±2.08[bc]
**Evenness (*E*)**	0.70±0.01[a]	0.79±0.00[b]	0.88±0.01[c]	0.89±0.02[c]

[a-c]Means that different letters within a row indicate a significant difference ($P < 0.05$) between them.
