# Supplementary figures and images for: Alteration of intestinal microflora by the intake of millet porridge improves gastrointestinal motility
Source: Front Nutr. 2022 Aug 22;9:965687. doi: 10.3389/fnut.2022.965687 (PMC9442030; doi:10.3389/fnut.2022.965687)

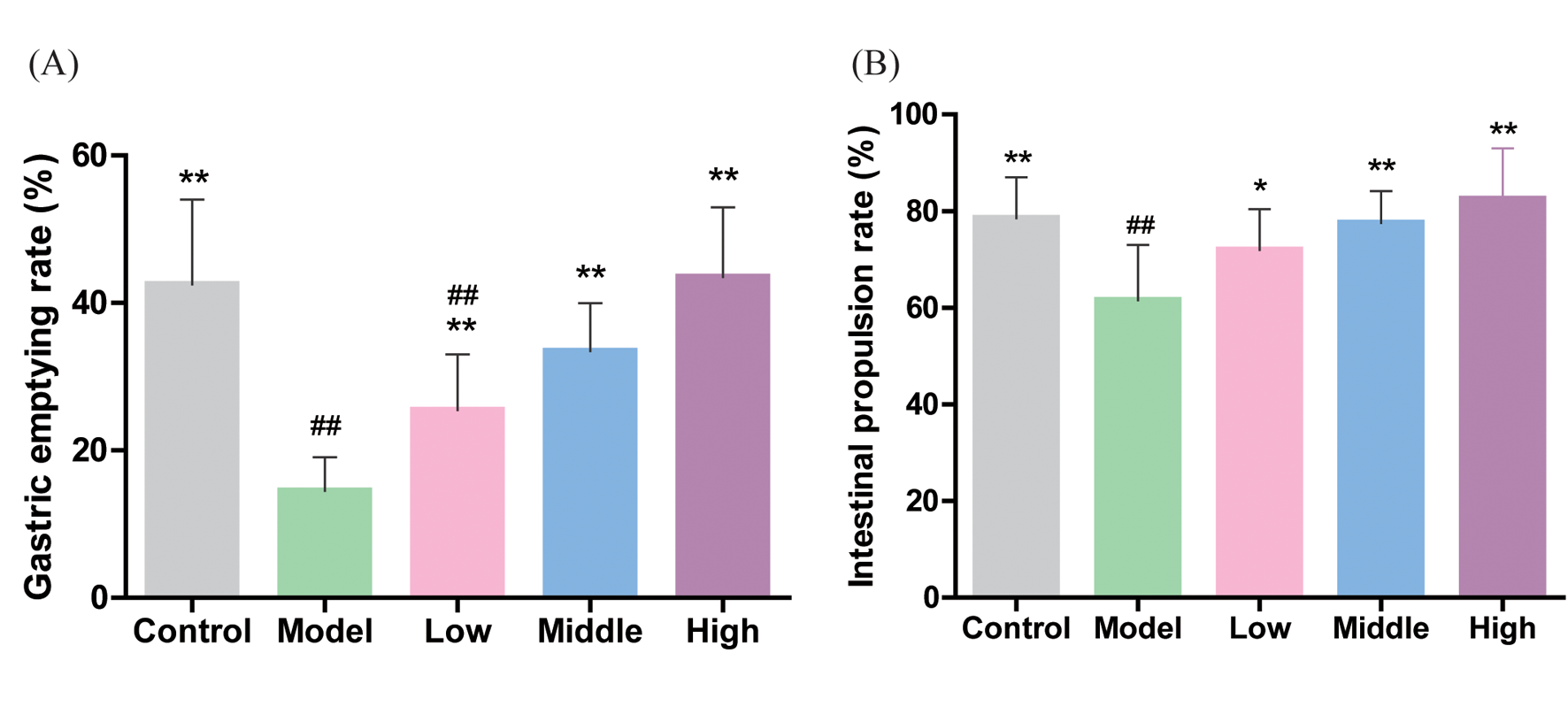

Supplement: Supplementary Figure 1 — Effect of millet porridge on gastric emptying (A) and intestinal propulsion (B). Diphenoxylate was used to induce the constipation model. The data are means ± SD (n = 10 for each group), ##P < 0.01 vs. control; *P<0.05, **P < 0.01 vs. Model. [file Image_1.TIF]
